# Mechanisms Used by Probiotics to Confer Pathogen Resistance to Teleost Fish

**DOI:** 10.3389/fimmu.2021.653025

**Published:** 2021-04-27

**Authors:** Rocío Simón, Félix Docando, Noelia Nuñez-Ortiz, Carolina Tafalla, Patricia Díaz-Rosales

**Affiliations:** Fish Immunology and Pathology Laboratory, Animal Health Research Centre (CISA-INIA), Madrid, Spain

**Keywords:** probiotics, pathogen resistance, interference mechanisms, immunomodulatory effect, teleosts

## Abstract

Probiotics have been defined as live microorganisms that when administered in adequate amounts confer health benefits to the host. The use of probiotics in aquaculture is an attractive bio-friendly method to decrease the impact of infectious diseases, but is still not an extended practice. Although many studies have investigated the systemic and mucosal immunological effects of probiotics, not all of them have established whether they were actually capable of increasing resistance to different types of pathogens, being this the outmost desired goal. In this sense, in the current paper, we have summarized those experiments in which probiotics were shown to provide increased resistance against bacterial, viral or parasitic pathogens. Additionally, we have reviewed what is known for fish probiotics regarding the mechanisms through which they exert positive effects on pathogen resistance, including direct actions on the pathogen, as well as positive effects on the host.

## History and Definition of Probiotics in Aquaculture

The term “probiotic” comes from the Latin word “pro” (for) and the Greek word “bios” (life) meaning “for life” (1) and it refers to microbial feed additives which confer a health benefit to the host organism through the modulation of intestinal microbiota. This first definition provided the basis of differentiating probiotics from antibiotics. The term “probiotics” was first proposed by Lilly and Stillwell (2) as “substances secreted by a micro-organism that stimulate the growth of another organism”, being substances microbially produced “factors”. Later on, Parker (3) was the first who defined probiotics as “organisms and substances which contribute to intestinal microbial balance”. As new findings emerged, the definition of “probiotic” was modified over the years. In 1989, Fuller defined probiotics as “live microbial feed supplements which beneficially affect the host animal by improving its intestinal microbial balance” (4), where the use of live microorganisms is emphasized, and the use of the word “substances” is removed, avoiding confusion. To accommodate the immunostimulatory effect of probiotics, Naidu et al. (5) modified the concept of probiotics as “microbial dietary adjuvants that beneficially affect the host physiology by modulating mucosal and systemic immunity, as well as improving nutritional and microbial balance in the intestinal tract”. Since then, many variations to the definition have still been proposed (6). The Food and Agriculture Organization of the United Nations/World Health Organization (FAO/WHO) integrated all these definitions and stated that probiotics are “live microorganisms, which when administered in adequate amounts confer a health benefit on the host” (7).

The first reported use of probiotics in aquaculture was in 1986 by Kosaza who evaluated the use of *Bacillus toyoi* spores as feed additives to increase the growth rate of yellowtail, *Seriola quinqueradiata* (8). But it was not until the late 1990s that research on probiotics became prominent in aquaculture. Given the fact that the aquatic animals constantly interact with their surrounding water environment, Moriarty (9) widened the definition of probiotics, also considering them as microbial “water additives”. Later on, Verschuere et al. (10) put forward the concept of aquaculture probiotics, proposing a broader application of the term as “live microbial adjuncts which have a beneficial effect on the host by modifying the host-associated or ambient microbial community by ensuring improved use of the feed or enhancing its nutritional value, by enhancing the host response towards diseases, or by improving the quality of its environment”. This definition allowed a wider application of the term “probiotic” by involving the aquatic environment.

The outmost desired goal of a probiotic is to have a positive effect on the general health status of the fish, thus increasing its resistance to pathogens. This can be achieved through different mechanisms, reviewed in the current paper, that cover direct interference with pathogens as well as effects on the host. Additionally, we have summarized those probiotics that have been shown to confer resistance against different types of pathogens, including bacteria, viruses and parasites.

## Source and Selection Criteria of Probiotics in Aquaculture

In the last decades, several microorganisms have been experimentally identified, characterized and applied in aquacultured species as probiotics. Probiotics tested for these species include a wide range of bacteria (Gram-negative or Gram-positive), yeasts, microalgae and bacteriophages which have been added to the water or included as feed supplements (11–17). Nevertheless, the list of probiotics commercially available for use in aquaculture is much more limited (18).

The source from where the probiotic microorganism is obtained varies greatly, including for example, the intestine of healthy fish, water of rearing environment, sediments of culture tanks, other animals or fermented food products (19). Because the main principle of a probiotic is to establish a relation with beneficial and harmful bacteria usually present in fish intestine, the gastrointestinal tract (20, 21) and the mucus (22) of aquatic animals are usually the most common sources to isolate microorganisms which can be used as potential probiotics. Although the probiotics could also have an origin outside the host, host-derived microorganisms are preferred given that microbiota living in healthy hosts can be considered part of the natural defense system, being beneficial to the host in multiple ways (23, 24). Furthermore, probiotics indigenous to the environment are able to survive spontaneously and function physiologically at their optimum level (21). It has to be taken into account, that, in contrast to terrestrial animals, the gastrointestinal microbiota of aquatic species is strongly dependent on the external environment due to the continuous water flow through the digestive tract. Hence, most of bacteria that colonize the tract are transient and could vary if the environmental conditions change (25).

In recent years, a large number of scientific works have been published regarding the screening, selection and characterization of fish probiotic bacterial strains (26–35). Potential candidates isolated from different sources are subjected to screening through multiple steps in order to assess their potential as ideal probiotics. Their safety (10) and lack of pathogenicity (36) have to be demonstrated as an essential first step. Thereafter, a successful probiotic candidate should meet certain criteria. Merrifield et al. (37) proposed an extended list of criteria, classifying them as either essential or favorable. As new findings emerged over the last decades, additional criteria have been added (12, 14). Taking all of this into account, the essential criteria to consider a microorganism as a suitable probiotic are the following: not being pathogenic, not only with regards to the host species, but also with regards to aquatic animals in general and human consumers; being free of plasmid-encoded antibiotic resistance genes; having the ability to tolerate a wide range of pH (low acidic to high alkaline) and high concentration (>2.5%) of bile salts. On the other hand, the merely favorable characteristics include: being able to adhere to and/or grow well within the intestinal mucus; being able to colonize the intestinal epithelial surface; being registered for use as a feed additive; displaying advantageous growth characteristics (e.g. short lag period, a short doubling time and/or growth at host rearing temperatures); exhibiting a broad spectrum of antagonistic activity against one or more key pathogens; producing relevant extracellular digestive enzymes or vitamins; being indigenous to the host or the rearing environment; remaining viable under normal storage conditions and being robust enough to survive industrial processes; having good sensorial properties, fermentative action, tolerance towards freeze-drying and viability in feed during packaging and storing process; having a beneficial effect on the growth, stimulation of immunity and protection of fish against various pathogenic bacteria. Although it is unlikely to find a candidate that will fulfill all of these characteristics, the more of these characteristics are fulfilled, the more likely it will be a promising probiotic. However, the main driver to select potential probiotics among different candidates has been their inhibitory activity against target pathogens *in vitro* (10, 28, 30, 38–40) or *in vivo* (11, 14, 15, 21, 41).

## Mechanisms of Interference of Probiotics With Pathogens

### Production of Inhibitory Substances

The antagonistic action or the inhibition of a variety of pathogens is one of the most important sought properties for potential probiotics. Probiotic microorganisms often have the capacity to produce substances which have bacteriostatic or bactericidal impact on pathogenic microbes, such as lysozymes, proteases, siderophores, hydrogen peroxide or bacteriocins (9, 42–50). For example, a compound named indole (2,3-benzopyrrole) with potent inhibitory activity against bacteria and fungus has been identified in some probiotic bacteria (51, 52). Similarly, some microorganisms produce volatile fatty acids (acetic, butyric, lactic and propionic acid) and organic acid, decreasing the gastrointestinal lumen’s pH, thereby preventing the proliferation of opportunistic pathogens (10, 47, 53–55).

In aquaculture, some candidate probiotics have been shown to produce antibacterial substances that inhibit the growth of harmful microbes and maintain intestinal microecological balance (43, 49). Thus, several probiotics used in aquaculture have been documented to exert direct antibacterial activities against known pathogens (14, 17). On the other hand, while knowledge on antiviral activity of probiotics has increased in recent years (14, 17), the exact mechanism of action through which these probiotic bacteria produce their antiviral effects remains still unknown. Yet, some studies performed *in vitro* revealed that the inhibition of viruses can occur through the action of extracellular enzymes secreted by the bacteria (14, 17). Finally, only a few studies have been reported the antifungal properties of fish probiotics (14, 17).

### Competition for Nutrients and Available Energy

All organisms, including bacteria, require a continuous source of nutrients for survival, growth and proliferation. Probiotics consume the available nutrients, thus, competition for nutrients is one of the mechanisms through which probiotics can inhibit pathogens (56). In fact, the survival of a microorganism will mainly depend on its potential to compete for nutrients and energy with other microorganisms in the same environment (10).

Among nutrients, iron is recognized to be the most important element, as it is an essential cofactor for important cellular processes, being required for DNA replication, oxygen transport, protection against oxidative stress, enzyme activity and energy generation (57). Thus, the majority of bacteria need iron for their growth, but the amount of iron available in animal tissues and body fluids is very limited. As a consequence, the competition for this nutrient between pathogenic bacteria and the host is a very well-known process (58). Siderophores are ferric ion specific chelators that are able to dissolve precipitated iron or extract it from iron complexes under iron-stressed conditions, making it available for bacterial growth (59). Siderophores are produced by several bacteria and fungus (59). Thus, the ability to produce siderophores is a favorable characteristic of a microorganism to be considered as a potential probiotic, in an iron-limited environment, as the probiotic would sequester ferric ion making it unavailable for the growth of pathogenic bacteria (60).

Siderophore production of fish probiotic strains has been investigated to some extent (61). Thus, for example, Smith and Davey (62) and Gram et al. (63) reported the inhibition of the growth of *Aeromonas salmonicida* and *Vibrio anguillarum*, respectively, under iron-limited conditions by *Pseudomonas fluorescens.* Similarly, Lazado et al. (64) showed the capacity of two bacterial isolates (GP21 *Pseudomonas* sp.; GP12 *Psychrobacter* sp.) obtained from the intestinal tract of Atlantic cod (*Gadus morhua*) to release siderophores, showing antagonistic activity against *V. anguillarum* and *A. salmonicida*. Also, the probiotic effect of a strain of *Vibrio* sp. has been associated with its capacity to compete for iron with a pathogenic strain of *Vibrio* sp. in seabass larvae (Dicentrarchus labrax) (65).

### Competition for Colonization of Mucosal Surfaces

The mucosal surface of fish is continuously interacting with the microbiota and the external media in an aquatic environment. In this context, pathogens invade the host through these mucosal surfaces, colonizing them and eventually spreading throughout the host and causing disease (66). Thus, most pathogenic bacteria need to attach to the mucosal layer of the host gastrointestinal tract (or other mucosal tissues) to exert a harmful effect and develop an infection (67).

In this sense, the ability of a microorganism to colonize and adhere to the epithelial surface, interfering with the pathogen’s adhesion is a favorable characteristic for the selection of candidate probiotics (37). In fact, competition for adhesion receptors with pathogens can be considered as an essential probiotic characteristic (68). Probiotics occupy the binding sites of the intestinal mucosa, forming a physical barrier, preventing the attachment of pathogenic microorganisms. Attachment of probiotics may be non-specific, based on physicochemical agents, or specific, based on the interaction of surface receptors on the adherent bacteria to receptor molecules on epithelial cells (19, 69). The mechanism through which a probiotic competes for adhesion sites is referred to as “competitive exclusion” (17).

Various authors have reported the ability of candidate fish probiotics to adhere to the host gastrointestinal tract and to interfere with pathogenic bacteria (66, 70–72). The interference of four potential probiotics (members of *Vibrionaceae* and *Pseudomonodaceae* families, as well as *Micrococcus* genus) with the pathogens *Listonella anguillarum* and *Vibrio harveyi* through competition for adhesion to the skin, gill and intestinal mucus of gilthead seabream (*Sparus aurata*) and Senegalese sole (*Solea senegalensis*) was demonstrated *in vitro* by Chabrillón et al. (73, 74). Furthermore, the *in vivo* probiotic potential of one of the selected candidates (Pdp11, *Vibrionaceae*) was assessed by oral administration and its ability to reduce the mortality after a challenge in gilthead seabream and sole against *L. anguillarum* and *V. harveyi* demonstrated, highlighting the relevance of this probiotic capacity. Another *in vitro* study investigated the potential of two candidate probiotic bacteria (GP21 and GP12) to adhere to primary cultures of epithelial cells obtained from different segments of the intestine and to interfere with the adhesion of two pathogens, *V. anguillarum* and *A. salmonicida* subsp. *salmonicida* in Atlantic cod. The study concluded that the adhesion of probiotics is segment-specific and the interference with the pathogen adhesion is dependent on both the source of epithelial cells and the mechanism through which the probiotic adheres to the epithelial cells (70). Through an *in vivo* study, Divya et al. (75) also confirmed the ability of three probiotic strains (*Bacillus coagulans*, *Bacillus mesentericus* and *Bifidobacterium infantis*) to colonize the gut of rosy barb (*Puntius conchonius*). This probiotic administration significantly changed the proportion of the gut microflora, decreasing the level of pathogenic strains. Vine et al. (72) reported the *in vitro* suppression of bacterial pathogen growth (*Aeromonas hydrophila* and *Vibrio alginolyticus*) as a consequence of their displacement by different probiotic candidate isolates that adhered to the intestinal mucus of spotted grunter (*Pomadasys commersonnii*). Similarly, the capacity of endogenous microbiota, e.g. *Lactobacilli*, to compete with pathogens for adhesion sites on the intestinal surface has also been established (76).

From a practical point of view, whether the applied probiotic is able to colonize the gut and for how long is a key issue to establish its administration regime (administration route, concentration and time of administration) and to provide farmers with a specific protocol with beneficial effects on fish health.

### Disruption of Quorum Sensing

Quorum sensing (QS) is the regulation of gene expression in response to fluctuations in cell-population density (77). QS is a regulatory mechanism by which the majority of bacteria communicate with each other and response collectively. To this end, bacteria synthesize and secrete small chemical signal molecules called auto-inducers whose concentration can be recognized by other bacteria, and in this way, perceive the surrounding cell density. Gram-negative bacteria secrete acyl-homoserine lactones (AHLs) as auto-inducers, while Gram-positive bacteria use oligopeptides. Both Gram-negative and Gram-positive bacteria can produce autoinducer-2 (AI-2). When a critical threshold concentration is achieved, the QS induces or represses the expression of genes involved in specific physiological functions (77, 78), including luminescence, virulence, motility, sporulation and biofilm formation (79–83).

As pathogenicity is controlled by QS, inhibiting this mechanism is a good strategy to control microbial pathogens. Thus, the disruption of QS is considered a potential anti-infective strategy in aquaculture (17, 84, 85). Quorum quenching (QQ), the disruption of QS, can be performed by molecule antagonists (86) or degrading enzymes (87). Thus, the QQ microorganisms can be used as potential quenchers of quorum-sensing-regulated functions in pathogenic bacteria (88, 89), acting as an alternative to antibiotics in the control of infections in aquatic systems. In aquaculture, QQ has also been demonstrated as an alternative to antibiotic control of infections (90, 91). In this context, probiotic bacteria with QQ capacities would be on one hand efficacious to control antibiotic-resistant pathogens while having other beneficial effects on the host (92). Along this line, searching for probiotics isolated from the intestinal microbiota of olive flounder (*Paralichthys olivaceus*), Zhang et al. (93) identified AHL lactonase (FiaL) in the genome of *Flaviramulus ichthyoenteri*. This FiaL degraded some signals used by different fish pathogens such as *A. hydrophila*, *Edwardsiella tarda*, *Vibrio salmonicida* and *V. anguillarum*; revealing a great potential of *F. ichthyoenteri* as a fish probiotic. Other studies reported the ability of some microorganisms to produce QS antagonists, such as halogenated furanones, which are produced by the marine red alga *Delisea pulchra* (94). These compounds were reported to protect *Brachionus*, *Artemia*, and rainbow trout (*Oncorhynchus mykiss*) from the negative effects of pathogenic *Vibrio* species (95, 96).

Other probiotic bacteria such as *Lactobacillus*, *Bifidobacterium* and *Bacillus cereus* strains degrade the signal molecules of pathogenic bacteria by enzymatic secretion or production of autoinducer antagonists (76). Thus, *Bacillus* sp. QSI-1 has been shown to significantly reduced the pathogenicity of *A. hydrophila* in *Carassius auratus gibelio* (84), zebrafish (*Danio rerio*) (79) and goldfish (*Carassius aurata*) (97) by degrading AHLs. Likewise, Ren et al. (98) reported the inhibition of growth and virulence of *A. hydrophila* by *Bacillus subtilis* involving QS. Another *Bacillus* species, *Bacillus licheniformis*, protects against *A. hydrophila* in zebrafish through QQ (99). In rainbow trout, Delshad et al. (100) established the QQ activity of different isolates (*B. cereus*, *Bacillus thuringiensis*, *Stenotrophomonas moltiphilia*, *Enterobacter hormaechei* subsp. *hormaechei* and *Citrobacter gillenii*), regulating the virulence of *Yersinia ruckeri*.

A recent publication focused on the isolation of autochthonous AHL degrading bacteria from the gastrointestinal tract of different fish species. Thus, Ghanei-Motlagh et al. (101) isolated several strains with beneficial QQ AHL-degrading and probiotic activities for the first time in Asian seabass (*Lates calcarifer*). Vadassery and Pillay (92) also focused at isolating AHL degrading bacteria from the gastrointestinal tract of Nile tilapia (*Oerocrhomis niloticus*). Among the isolated strains, *Enterococcus faecium* contained an autoinducer inactivation homolog gene with the ability to degrade N-AHL (N-acyl homoserine lactone) produced by the fish pathogen *A. hydrophila.*

## Immunomodulatory Effects of Probiotics

Probiotics have been shown to have the capacity to increase innate and adaptive immunity of fish, being the effects exerted on the fish innate immune system the main desirable characteristics of candidate probiotics (102). Probiotics can influence both the systemic and the local immunity of the host when they are administered i) orally or through the rearing water, or ii) as live or as dead cells (102). In some studies, the immunomodulatory effect of probiotics was attributed to the release of cytokines, key regulators in orchestrating the immune response in fish, which include interleukins (ILs), tumor necrosis factors (TNFs), interferons (IFNs), transforming growth factors (TGF) and chemokines from immune cells such as lymphocytes, granulocytes, macrophages, mast cells, epithelial cells, and dendritic cells (DCs) (103, 104). In this review, we report some of the immunomodulatory effects that probiotics have been shown to exert on the mucosal immune system.

As mentioned above, fish are constantly interacting with their surrounding water environment. In this sense, mucosal tissues are strategically located in areas where environmental pathogens enter the body. Thus, the mucosal immune system has a pivotal role in the defense mechanism against pathogens and thus considered as a very active immunological site (105). The mucosal surfaces of the fish include the epithelia and the mucosa-associated lymphoid tissues (MALTs). The main MALTs in teleost fish include: GALT (gut-associated lymphoid tissue), SALT (skin-associated lymphoid tissue), GIALT (gill-associated lymphoid tissue) and NALT (nasopharynx-associated lymphoid tissue). All teleost MALTs have common features: i) the presence of a mucus layer, that envelops the majority of the epithelia and consisting mainly of high molecular weight glycoproteins called mucins secreted by the epithelial globet cells. This mucus layer acts as a physical and chemical barrier preventing the entry of pathogens; ii) the presence of innate and adaptive immune components, such as cytokines or immunoglobulins (Igs), among many others; iii) the transport of antibodies across the epithelium by the polymeric Ig receptor (pIgR); and iv) the presence of a complex and diverse commensal bacterial community (microbiota) that plays a critical role in maintaining the host’s physiology homeostasis. In contrast to mammals, the intestinal immune system of fish lacks lymphoid tissue aggregates such as the Peyer’s patches, instead they have a diffuse GALT, and the inductive and effector sites of the lamina propria can hence not be distinguished from each other. However, similar to higher vertebrates, GALT contains mucosal immune cells such as lymphocytes, plasma cells, granulocytes and macrophages present in the epithelium or distributed in the lamina propria (105–107) and these potentiate this mucosal tissue as an active immune organ.

Many studies have demonstrated that probiotic supplementation influences the GALT by modulating gut morphology and the population of intestinal immune cells as well as their physiological activities (102, 108). In addition, probiotics could also manipulate the richness and diversity of the commensal gut microbiota, which in turn may interact with pathogens to influence their success in the intestine. However, to date, despite the great advances made in this field in the past years, there are still many gaps regarding our understanding of how microbiota composition influences mucosal responses in teleosts.

Lactic acid bacteria (LAB) and *Bacillus* spp. are among the most commonly used probiotic candidates in aquaculture (108, 109). Thus, several effects in the gut immune system have been reported upon LAB administration in different fish species. For example, the administration of *Lactobacillus rhamnosus* in Nile tilapia resulted in increased villous height in the proximal and mid intestine as well as increased intraepithelial lymphocytes numbers and acidophilic granulocytes (110). In an earlier study performed in rainbow trout, the co-administration of *Lactococcus lactis* subsp*. lactis*, *Leuconostoc mesenteroides* and *Lactobacillus sakei* resulted in an enhanced phagocytic activity of gut leukocytes (111). Other direct effects on the gut immune system that have been observed in LAB-fed fish include: stimulation of pro-inflammatory cytokines such as IL-1, IL-6, IL-2, TNF-α and IFN-γ and also anti-inflammatory cytokines such as IL-10 and TGF-β; increased gene expression of immune-related genes such as MHC II or IgM; increased presence of T cells; increased mucin-secreting goblet cell numbers; increased total Ig concentration (112–117). Certainly, all these probiotic immune effects vary depending on the types of LAB administered and on the host species, but in general are mostly immunostimulatory pro-inflammatory effects. It is interesting to note that, in contrast, the effects of probiotics on the gut immune system in mammals are mostly anti-inflammatory (118). In mammals, probiotics have been seen to provoke anti-inflammatory effects indirectly by maintaining or repairing epithelial barriers, enhancing the production of short chain fatty acids with anti-inflammatory properties or by inducing the synthesis of antimicrobial peptides that influence inflammation resolution in the mucosa. Also in mammals, probiotics bind innate immune receptors and trigger pathways that affect the production of both pro- and anti-inflammatory cytokines. Despite the capacity to induce both types of cytokines, the overall balance is generally anti-inflammatory. Hence, although the reason for this discrepancy between the effects that probiotics have on inflammation in fish and mammals is currently unknown, it seems obvious that the immunomodulatory properties of a given probiotic are not only dependent on the inherent features of the microorganism used but also on the complexity of the immune system of the host.

Regarding *Bacillus* spp., numerous investigations have demonstrated their efficacy and potency as probiotics in aquaculture (109). In an overview, *Bacillus* probiotics have been shown to have the capacity to modulate some innate immune responses such as phagocytic and lysozyme activity, respiratory burst, antiprotease and peroxidase, superoxide dismutase and myeloperoxidase through effects on different some immunocompetent cell populations. These probiotics have also been shown to generate changes in the physiology of immune cells, for example, increasing of neutrophil adherence capacity, neutrophil migration and plasma bactericidal activity that in the end can result in the improvement of immune effector functions such as enhancement in complement activity, Ig production and cell cytotoxicity (119–121). All these immune-stimulatory effects exerted by *Bacillus* occur in the GALT, although further research work is needed to understand the detailed mechanisms.

The mucus is a key element of mucosal immunity, thus, some studies have focused on determining how probiotics affect the mucus layer in different ways. Hence, for example, Cerezuela and collaborators extensively studied the effects of diets enriched with two different probiotics, *Shewanella putrefaciens* and *Bacillus* sp. on the skin mucus of gilthead seabream (122). Both probiotics were shown to significantly alter the carbohydrate composition of the mucus, its IgM content and its enzymatic activity, with some differences depending on the probiotic used. In the case of Nile tilapia, feed supplementation with *Lactobacillus plantarum* (123) or *Bacillus licheniformis* (124) was shown to increase the enzymatic activity of the skin mucus, whereas diets containing different *Bacillus* strains significantly augmented its nitric oxide (NO) and IgM content, and lysozyme and alkaline phosphatase activity (125). The protein profile of the skin mucus was also altered in Crucian carp (*Carassius auratus gibelio*) fed *Bacillus cereus* (126) or *Lactobacillus acidophilus* (127). Despite their relevance, the number of studies that have investigated the effects of probiotics on the intestinal mucus is much more reduced. In this sense, some studies have reported a significant increase in the number of goblet cells in the intestinal mucosa (125, 128) or an increased IgM content of the intestinal mucus (128) in response to a prolongued administration of probiotics.

Finally, it has to be mentioned that many other studies have addressed the systemic immune effects of fish probiotics, but due to length restrictions of this review, we will not refer to them in depth. Most of these studies have been focused on describing increased serum IgM levels (129), increased humoral innate immune parameters or transcriptional changes in systemic immune tissues (reviewed in 129–131) upon probiotic treatment.

## Additional Effects of Probiotics on Fish

### Production of Beneficial Substances

Probiotics can also produce some substances with beneficial effects, which are useful to the host for feed conversion, growth performance and immunity. Thus, the capability of a microorganism to produce extracellular enzymes, such as proteases, amylases, cellulases, phytases, chitinases, lipases, etc., is also a desirable characteristic of a probiotic candidate. Fish produce a wide range of endogenous enzymes such as those listed above (132–135), however, their quantity and activity are not adequate for a complete metabolism of the ingested materials from feed. Thus, enzymes secreted by permanent gut endosymbionts and potential probiotics are essential from a nutritional perspective (136), contributing to the digestive process of the host. In recent years, the capacity of several fish probiotic strains to produce extracellular enzymes has been extensively investigated (12, 16). For example, Dawood et al. (137) reported that *Lactobacillus plantarum* significantly enhanced amylase, lipase and protease activity of Nile tilapia. Supplementation of olive flounder with this probiotic (*L. plantarum*) as well as with *Bacillus* sp. increased several enzyme activities such as amylase, trypsin and lipase (138). Significant increase of theses enzymes, together with proteases was also reported in carp (*Cyprinus carpio*) after the administration of *Lactobacillus casei* in combination with β-glucan and mannan oligosaccharide (139). Other LAB, *Lactobacillus bulgaricus* and *Lactobacillus acidophilus*, together with *Citrobacter* were reported to increase amylase, trypsin and alkaline phosphatase in rainbow trout (140). Tarkhani et al. (141) described the increase of intestinal digestive enzyme activities of Caspian roach (*Rutilus caspicus*) after the administration of *E. faecium*. Despite of the reported results, the actual contribution of these enzymes to the fish metabolism is still not well understood.

In general, fish do not produce any vitamins and endosymbionts/probiotics are the primary producers of vitamins, making them available to the host. Thus, many probiotics have been shown to supply vitamins, fatty acids and essential amino acids to the host (45, 111, 142, 143). Besides bacterial probiotics, many strains of yeast have been used as dietary supplements in a number of fish species (144). Interestingly, yeasts can produce polyamines, which enhance intestinal maturation (145). Therefore, considering the provision of vital nutrients such as fatty acids, biotin and vitamins, probiotics might be also considered as a complementary food source (10).

### Promotion of Growth Performance

As probiotics contribute to improve the feed consumption and nutrient’s uptake, they also have positive effects on the host growth rate (146). Thus, probiotics often lead to an enhanced growth performance, as well as an increased survival rate.

*Lactobacillus* is the most studied genus of bacteria regarding its effects on growth performance. Dietary administration of *L. plantarum* enhanced growth parameters of several fish species (carp, Nile tilapia, brown trout, *Salmo trutta caspius*; 123, 137, 147–150). Furthermore, the combination of *L. plantarum* with other probiotics and natural immunostimulants was also shown to increase of growth rate in different fish. Thus, Alishahi et al. (151) reported an increase in the weight gain of carp after dietary administration of a combination of *L. plantarum* with *L. bulgaricus*. The growth performance of Nile tilapia was increased after administration of *L. plantarum* together with the fungus *Cordyceps militaris* (152), and the catfish (*Pangasius bocourti*) with artichoke (153) or with *Bacillus velezensis* (154). *L. lactis* is another probiotic whose positive effect on growth performance has been reported in several farmed fish species, when administered alone (155–158); in combination with immunostimulants, such as β-glucan and mannan oligosaccharide (139); or other *Lactobacillus* (117). The ability to increase the growth rate has been demonstrated for other species of *Lactobacillus*, such as *Lactobacillus delbrueckii* (159), *L. rhamnosus* (117, 160), *L. bulgaricus*, *L. acidophilus* (138), and for other bacteria species, such as *Citrobacter* in combination with *L. bulgaricus* and *L. acidophilus* (140), *Pediococcus* (161, 162) and *Enterococcus* (141, 163). For example, Asian seabass increased its growth after the administration of a commercial probiotic consisting in *Lactobacillus* spp.*, E. faecium, B. subtilis and Saccharomyces cerevisiae*. *Streptoccocus faecium* in combination with *L. acidophilus* and *S. cerevisiae* was also reported to act as a growth promoter for Nile tilapia (164, 165).

The role of *Bacillus* probiotics as growth promoters has been reported in several farmed fish species. Thus, dietary administration of *B. subtilis* enhances growth of Nile tilapia (164, 166), carp (167) and grass carp, *Ctenopharyngodon idella* (168). *B. subtilis* has been administered in combination with *L. lactis* and increase the growth of rohu, *Labeo rohita* (169), as well as *Peidococcus acidilactici* in rainbow trout (170). Growth of catfish was increased due to the administration of *Bacillus amyloliquefaciens* and *Bacillus pumilus* (171). Similarly, *B. coagulans* enhanced the growth of carp (172) and *B. licheniformis* functioned as a growth promoter in tilapia (173).

## Established Effects of Probiotics on Pathogen Resistance

The use of probiotics in aquaculture is still faced with a lot of controversies and skepticism. However, the capacity of probiotics to increase the resistance of fish against different types of pathogens, including bacteria, viruses and parasites, has been widely demonstrated experimentally by higher survival rates upon pathogen challenge in probiotic-treated fish when compared to controls (15, 56, 108). Thus, in this section, we briefly review all the published data concerning increased resistance of aquacultured fish to bacteria, viruses or parasites upon probiotic treatment. The main information regarding these studies has been also summarized in **Table 1** (bacteria), **Table 2** (viruses) and **Table 3** (parasites).

**Table 1:** Probiotics assayed *in vivo* in aquacultured species which have shown to confer significant resistance against bacterial pathogens.

Fish species	Probiotic	Probiotic administration route	Pathogen	Pathogen administration route	Reference
*A. anguilla*	*E. faecium* and *B. toyoi*	Diet	*E. tarda*	Anal	(174)
*A. japonica*	*L. pentosus*	Diet	*E. tarda*	i.p.	(175)
*C. auratus* and *X. helleri*	*Lactobacillus* sp., *Bacillus* sp. and commercial aquaculture probiotic (CA probiotic)	Diet	*P. fluorescens*	IMM	(176)
*C. auratus gibelio*	*Bacillus* sp.	Diet	*A. hydrophila*	i.p.	(177)
*C. macrocephalus* x *C. gariepinus* (hybrid)	*B. siamensis*	Diet	*A. hydrophila*	i.p.	(178)
*C. gariepinus*	*L. acidophilus*	Diet	*S. xylosus*, *A. hydrophila* and *S. agalactiae*	i.p.	(179)
*C. catla*	*B. circulans*	Diet	*A. hydrophila*	IMM	(180)
*C. altivelis*	*L. lactis*	Diet	*V. harveyi*	i.p.	(158)
*C. idellus*	*S. xiamenensis* and *A. veronii*	Diet	*A. hydrophila*	i.p.	(181)
*C. idella*	*P. pentosaceus*	Diet	*A. hydrophila*	i.p.	(182)
*C. carpio*	*A. veronii*, *V. lentus* and *F. sasangense*	Diet	*A. hydrophila*	i.p.	(183)
*C. carpio*	*L. plantarum*	Diet	*A. hydrophila*	i.p.	(148)
*C. carpio*	*L. delbrueckii*	Diet	*A. hydrophila*	i.p.	(159)
*C. carpio*	*L. lactis*	Diet	*A. hydrophila*	i.p.	(155)
*D. labrax*	*V. fluvialis*	Diet	*V. anguillarum*	i.p.	(184)
*D.labrax*	*E. gallinarum*	Diet	*V. anguillarum*	IMM	(185)
*E. coioides*	*L. plantarum*	Diet	*Streptococcus* sp.	i.m.	(186)
*E. coioides*	*P. pentosaceus*	Diet	*V. anguillarum*	i.p.	(187)
*Epinephelus* spp.	*S. cerevisiae*	Diet	*Streptococcus* sp.	i.m.	(188)
*G. morhua*	*C. divergens*	Diet	*V. anguillarum*	IMM	(189)
*G. morhua*	*C. divergens*	Diet	*V. anguillarum*	IMM	(190)
*G. morhua*	*P. gallaeciensis*	IMM	*V. anguillarum*	IMM	(191)
*L. rohita*	*B. subtilis*	Diet	*A. hydrophila*	i.p.	(192)
*L. rohita*	*B. subtilis*	Diet	*E. tarda*	i.p.	(193)
*L. rohita*	*B. subtilis*	Diet	*A. hydrophila*	i.p.	(194)
*L. rohita*	*P. aeruginosa*	Diet	*A. hydrophila*	i.p.	(195)
*L. rohita*	*L. plantarum*	Diet	*A. hydrophila*	i.p.	(196)
*L. rohita*	*Bacillus* spp.	Diet	*A. hydrophila*	i.p.	(197)
*L. crocea*	*B. subtilis*	Diet	*V. harveyi*	i.p.	(198)
*L. calcarifer*	*L. casei, L. plantarum, L. pentosus, L. fermentum*, *E. faecium* and *B. subtilis*	Diet	*A. hydrophila*	i.p.	(199)
*M. miiuy*	*C. butyricum*	Diet	*V. anguillarum* and *A. hydrophila*	i.p.	(200)
*M. rosacea*	*D. hansenii*	Diet	*A. hydrophila*	i.p.	(201)
*O. mykiss*	*C. butyricum*	Oral	*V. anguillarum*	i.p.	(202)
*O. mykiss*	*P. fluorescens*	IMM	*V. anguillarum*	IMM	(63)
*O. mykiss*	*Carnobacterium* sp.	Diet	*A. salmonicida*	CO	(203)
*O. mykiss*	*L. rhamnosus*	Diet	*A. salmonicida*	CO	(31)
*O. mykiss*	*Pseudomonas* spp. and *Carnobacterium* spp.	IMM	*V. anguillarum*	IMM	(204)
*O. mykiss*	*A. hydrophila, Vibrio* spp., *Carnobacterium* spp. and an unidentified Gram-positive coccus	Diet	*A. salmonicida*	CO, i.m., i.p.	(205)
*O. mykiss*	*A. hydrophila, V. fluvialis, Carnobacterium* spp. and an unidentified Gram-positive coccus	Diet	*A. salmonicida*	CO	(206)
*O. mykiss*	*B. subtilis* and *B. licheniformis*	Diet	*Y. ruckeri*	i.p.	(207)
*O. mykiss*	*A. sobria*	Diet	*S. iniae*	i.p.	(208)
*O. mykiss*	*C. maltaromaticum* and *C. divergens*	Diet	*Y. ruckeri* and *A. salmonicida*	i.p.	(209)
*O. mykiss*	*L. lactis* and *L. mesenteroides*	Diet	*A. salmonicida*	CO (asymptomatic carrier)	(38)
*O. mykiss*	*Bacillus* spp. and *A. sobria*	Diet	*A. salmonicida*, *L. garvieae*, *S. iniae*, *V. anguillarum*, *V. ordalii and* *Y. ruckeri*	i.p.	(210)
*O. mykiss*	*B. subtilis*	Diet	*Aeromonas* sp.	i.p.	(211)
*O. mykiss*	*A. sobria* and *B. thermosphacta*	Diet	*A. bestiarum*	i.m.	(212)
*O. mykiss*	*L. mesenteroides* and *L. plantarum*	Diet	*L. garvieae*	CO	(196)
*O. mykiss*	*E. cloacae* and *B. mojavensis*	Diet	*Y. ruckeri*	IMM	(213)
*O. mykiss*	*E. faecalis*	Diet	*V. anguillarum*	i.p.	(214)
*O. mykiss*	*Kocuria*	Diet	*V. anguillarum*	i.p.	(215)
*O. mykiss*	*Kocuria*	Diet	*V. anguillarum* and *V.ordalii*	i.p.	(216)
*O. mykiss*	*Enterobacter* sp. and *E. amnigenus*	Diet	*F. psychrophilum*	i.m.	(217)
*O. mykiss*	*Pseudomonas* sp.	Diet	*F. psychrophilum*	i.m.	(218)
*O. mykiss*	*Kocuria* and *Rhodococcus*	i.p.	*V. anguillarum*	i.p.	(219)
*O. mykiss*	*E. faecalis*	Diet	*A. salmonicida*	i.p.	(220)
*O. mykiss*	*Enterobacter* sp.	Diet	*F. psychrophilum*	i.p.	(221)
*O. mykiss*	*E. casseliflavus*	Diet	*S. iniae*	i.p.	(222)
*O. mykiss*	*E. faecalis*	Diet	*L. garvieae*	i.p.	(163)
*O. mykiss*	*L. delbrukei* subsp. *bulgaricus*, *L. acidophilus* and *C. farmeri*	Diet	*L. garvieae*	i.p.	(116)
*O. fasciatus*	*L. sakei*	Diet	*E. tarda*	i.p.	(223)
*O. mossambicus*	Lactic acid bacteria	Diet	*A. hydrophila*	IMM	(48)
*O. niloticus*	*L. rhamnosus*	Diet	*E. tarda*	i.p.	(224)
*O. niloticus*	*S. cerevisiae*	Diet	*A. hydrophila*	i.p.	(225)
*O. niloticus*	*B. subtilis* and *L. acidophilus*	Diet	*A. hydrophila*, *P. fluorescens* and *S. iniae*	i.p.	(226)
*O. niloticus*	*B. pumilus*	Diet	*A. hydrophila*	i.p.	(227)
*O. niloticus*	*M. luteus* and *Pseudomonas* spp.	Diet	*A. hydrophila*	i.p.	(228)
*O. niloticus*	*L. brevis* and *L. acidophilus*	Diet	*A.hydrophila*	i.p.	(229)
*O. niloticus*	*B. licheniformis*	Diet	*S. iniae*	i.p.	(230)
*O. niloticus*	*B. amyloliquefaciens*	Diet	*Y. ruckeri* and *C. perfringens*	i.p.	(231)
*O. niloticus*	*B. subtilis*	Diet	*S. agalactiae*	i.p.	(166)
*O. niloticus*	*B. pumilus*	Diet	*S. agalactiae*	i.m.	(232)
*O. niloticus*	*L. garvieae*	Diet	*S. aureus*	i.p.	(233)
*O. niloticus*	*L. plantarum* and *B. velezensis*	Diet	*S. agalactiae*	i.p.	(154)
*O. niloticus*	*L. rhamnosus* and *L. lactis* subsp. *lactis*	Diet	*S. agalactiae*	i.p.	(117)
*O. niloticus*	*L. plantarum*	Diet	*A. sobria*	i.p.	(234)
*O. niloticus*	*L. plantarum*	Diet	*S. agalactiae*	i.p.	(235)
*O. niloticus*	*L. plantarum*	Diet	*S. agalactiae*	i.p.	(123)
*Oreochromis* sp.	*B. subtilis*, *B. licheniformis*, *Bacillus* sp. and *Pediococcus* sp.	Diet	*S. agalactiae*	CO	(236)
*P. hypophthalmus*	*B. amyloliquefaciens* and *B. pumilus*	Diet	*E. ictaluri*	i.p.	(171)
*P. bocourti*	*B. aerius*	Diet	*A. hydrophila*	i.p.	(237)
*P. olivaceus*	*B. licheniformis*	Diet	*S. iniae*	IMM	(238)
*P. olivaceus*	*L. lactis*	Diet	*S. iniae*	CO	(239)
*P. olivaceus*	*L. lactis* and *L. plantarum*	Diet	*S. iniae*	i.p.	(240)
*P. olivaceus*	*L. lactis*	Diet	*E. tarda*	i.p.	(241)
*P. olivaceus*	*L. lactis*	Diet	*S. parauberis*	Oral	(156)
*P. fluviatilis*	*P. chlororaphis*	Diet	*A. sobria*	IMM	(242)
*R. canadum*	*B. subtilis*	Diet	*V. harveyi*	i.p.	(243)
*S. trutta*	*L. lactis* and *L. mesenteroides*	Diet	*A. salmonicida*	Asymptomatic carrier	(244)
*S. salar*	*P. fluorescens*	IMM	*A. salmonicida*	CO	(62)
*S. maximus*	*Roseobacter* spp.	IMM	*V. anguillarum*	IMM	(245)
*S. maximus* L.	*Roseobacter* sp.	Diet (bioencapsulated in rotifers)	*V. anguillarum*	Diet (bioencapsulated in rotifers)	(246)
*S. maximus*	*Bacillus* spp.	Diet	*V. anguillarum*	i.p.	(247)
*S. schlegeli*	*P. acidilactici*	Diet	*E. tarda*	i.p.	(162)
*S. senegalensis*	*S. putrefaciens* and *S. baltica*	Diet	*P. damselae* subsp. *piscicida*	i.p.	(248)
*S. senegalensis*	*S. putrefaciens*	Diet	*P. damselae* subsp*. piscicida*	i.p.	(249)
*S. aurata*	*Micrococcus* and *Vibrionaceae*	Diet	*L. anguillarum*	i.p.	(29)

i.p., intraperitoneal injection; i.m., intramuscular injection; IMM, immersion; CO, cohabitation.

**Table 2:** Probiotics assayed *in vivo* in aquacultured species which have shown to confer significant resistance against viral pathogens.

Fish species	Probiotic	Probiotic administration route	Pathogen	Pathogen administration route	Reference
*C. bartholomaei*	*P. undina*	IMM	SJNNV	n.d.	(250)
*C. carpio*	*L. plantarum* (expressing G protein from SVCV)	Oral	Spring Viremia of Carp Virus (SVCV)	Injection (n.d.)	(251)
*E. coioides*	*L. plantarum*	Diet	Grouper Iridovirus (GIV)	i.p.	(186)
*E. coioides*	*S. cerevisiae*	Diet	Grouper Iridovirus (GIV)	i.m., i.p.	(188)
*E. coioides*	*B. subtilis*	Diet	Iridovirus (GIV)	i.p.	(252)
*E. fuscoguttatus* x *E. lanceolatus* (hybrid)	*B. subtilis*	Diet	Singapore grouper iridovirus (SGIV)	IMM	(253)
*O. mykiss*	*L. casei* (expressing VP2 & VP3 from IPNV)	Oral	Infectious Pancreatic Necrosis Virus (IPNV)	i.p.	(254)
*O. mykiss*	*L. lactis* and *L. lactis* (expressing G protein from VHSV)	Diet	Viral haemorrhagic septicaemia virus (VHSV)	i.p.	(255)
*O. mykiss*	*L. casei* (expressing AHA1-CK6 and VP2 from IPNV	Diet	Infectious Pancreatic Necrosis Virus (IPNV)	i.p.	(246)
*P. olivaceus*	Commercial probiotics: Lactobacil and Sporolac (Inter Care Ltd, Mehsana, Gujarat)	Diet	Lymphocystis disease virus (LCDV)	Natural infection	(256)

i.p., intraperitoneal injection; i.m., intramuscular injection; IMM, immersion; n.d., not determined.

**Table 3:** Probiotics assayed *in vivo* in aquacultured species which have shown to confer significant resistance against parasites.

Fish species	Probiotic	Probiotic administration route	Pathogen	Pathogen administration route	Reference
*C. idellus*	*B. subtilis*	Diet	*C. sinensis*	CO	(257)
*C. carpio*	*Bacillus* spp., *Lactobacillus* sp. and *Nitrosomonas* sp.	IMM	*Myxobolus* sp.	Natural infection	(258)
*O. mykiss*	*A. sobria* and *B. thermosphacta*	Diet	*I. multifilis*	IMM	(212)
*P. hypophthalmus*	*L. plantarum*	IMM	*S. parasitica*	IMM	(259)

IMM, immersion; CO, cohabitation.

### Probiotics Providing Resistance to Bacterial Pathogens

Most of the investigations in the literature that have studied pathogen resistance conferred by probiotics have studied it in relation to bacterial pathogens.

As mentioned above, the most commonly used probiotic species in aquaculture include genera *Lactobacillus* and *Bacillus* (108, 260). In all the studies summarized in **Table 1**, apart from these probiotic species, there are other Gram-positive bacteria frequently used that include genera *Carnobacterium*, *Lactococcus*, *Leuconostoc*, *Pediococcus*, *Enterococcus*, *Clostridium*, *Micrococcus*, *Rhodococcus* and *Kocuria*. Regarding the Gram-negative bacteria, *Pseudomonas*, *Aeromonas*, *Shewanella*, *Enterobacter*, *Citrobacter*, *Roseobacter*, *Vibrio* and *Flavobacterium* have also been tested with positive effects. Regarding yeast, the genera *Saccharomyces* is the most commonly used. Interestingly, in numerous investigations the mixture of different probiotic candidates, mainly LAB or *Bacillus* spp. together or with other species (see **Table 1**) resulted in higher disease resistance against bacteria (also reviewed in 108). Indeed, as mentioned throughout the review, the combination of different probiotics and other immunostimulants resulted in higher positive effects on the host, not only regarding pathogen resistance but also on growth performance or in the immune response.

In almost all studies described in **Table 1**, the probiotic candidates were administered along with the diet. *Lactobacillus* spp. constitutes one of the probiotics for which antibacterial activity has been more frequently shown. Thus, the dietary supplementation of *L. rhamnosus* increased disease resistance of rainbow trout against *A. salmonicida* (31); Nile tilapia against *E. tarda* (224) and, in combination with *L. lactis*, also increased disease resistance of Nile tilapia against *Streptococcus agalactiae* (117). In case of dietary inclusion of *L. plantarum*, it has also been shown that significantly increased disease resistance of common carp and *L. rohita* against *A. hydrophila* (148, 196); rainbow trout against *Lactoccocus garvieae* (261); *Epinephelus coioides* against *Streptococcus* sp. (186) and Nile tilapia against *Aeromonas sobria* (234). Other bacteria species of genera *Lactobacillus*, such as *L. pentosus*, *L. acidophilus*, *L. fermentum*, *L. delbrueckii* or *L. casei* have also been studied as probiotic candidates improving disease resistance against a variety of bacterial pathogens, when administered with the diet alone or in combinations (see **Table 1**). Regarding *Lactococcus* spp., it has been shown that diet supplementation of these probiotic species also led to the improvement disease resistance of common carp against *A. hydrophila* (155); *Chromileptes altivelis* against *V. harveyi* (158); rainbow trout against *A. salmonicida* (38); olive flounder against *E. tarda* (241), *S. iniae* (239, 240) and also against *Streptococcus parauberis* (156); Nile tilapia against *Staphylococcus aureus* (233); *Oreochromis mossambicus* against *A. hydrophila* (48) and brown trout against *A. salmonicida* (244). In many other studies, *Carnobacterium* spp. were the selected microorganisms to be investigated as probiotic candidates and dietary administration of these bacteria species resulted in enhanced disease resistance of Atlantic cod against *V. anguillarum* (189, 190); and rainbow trout against *Y. ruckeri* and/or *A. salmonicida* (203, 209). Also, in some investigations, the mixture of these bacteria together with *A. hydrophila* and *Vibrio* spp., resulted in increased resistance of rainbow trout against *A. salmonicida* (203, 206, 262). Similar results were also revealed in numerous investigations where *Bacillus* spp. appears as the selected probiotic agent to study the control of fish disease (**Table 1**). For example, dietary supplementation of *B. subtilis* and *B. licheniformis* significantly increased disease resistance of rainbow trout against *Y. ruckeri* (207). Likewise, rainbow trout fed *Bacillus* spp. and *A. sobria* showed enhanced disease resistance against *S. iniae* (210), and *L. rohita* fed *B. subtilis* showed enhanced disease protection against *E. tarda* (193) and *A. hydrophila* (194, 197). In case of fish-fed with *Aeromonas* spp., increased disease resistance against *S. iniae and A. salmonicida* were shown (206, 208, 210, 262). Another study worth mentioning is that of Gong et al. (182), that isolated a new *Pediococcus pentosaceus* strain (SL001) which exhibited a wide antimicrobial spectrum against fish pathogens, including *A. hydrophila*, *Aeromonas veronii*, *A. sobria*, *E. tarda*, *L. garvieae*, and *Plesiomonas shigelloide*. Less frequent are the studies that used yeasts as probiotic agents; however, we found a few of them in the literature in which dietary supplementation with *S. cerevisiae* significantly increased the disease resistance of *Ephinephelus* spp. against streptococcosis (188) or Nile tilapia against *A. hydrophila* (225); in the same way, fish-fed *Debaryomyces hansenii* presented increased disease resistance against *A*. *hydrophila* (201). The effect of dietary inclusion of other selected probiotics, such as *Clostridium butyricum* or *Enterobacter cloacae*, to control fish disease against a variety of bacterial pathogens, such as vibriosis or yersiniosis, respectively, are also summarized in **Table 1**.

### Probiotics Providing Resistance to Viral Pathogens

Some studies searching for bacteria with antiviral activity have been carried out in fish, especially during the 80s and 90s (**Table 2**). In 1988, Kamei et al. performed a plaque reduction assay to screen the antiviral activity of bacteria isolated from fresh water salmonid hatcheries against infectious hematopoietic necrosis virus (IHNV). The results showed that different *Pseudomonas* spp. and *Aeromonas* spp. strains produced a 90% plaque reduction (263). In 1997, Maeda et al. performed a natural infection of the yellow jack (*Carangoides bartholomaei)* larvae with Sima-aji Neuro Necrosis Virus (SJNNV), reporting that the bacterial strain *Pseudoalteromonas undina* VKM-124 showed an inhibitory activity towards SJNNV (250), consequently increasing the survival rate of the yellow jack larvae. In another study, carried out by Son et al., the dietary administration of the probiotic *L. plantarum* enhanced disease resistance of the grouper *E. coioides* against grouper iridovirus (GIV). Interestingly, fish fed *L. plantarum* also showed enhanced growth and innate immune responses, such as respiratory burst or plasma lysozyme activity among other effects (186). In a similar way, Chiu et al., found that the dietary administration of the yeast probiotic, *S. cerevisiae* P13 isolated from fermented peaches, enhanced disease resistance of *E. coioides* against GIV (188). Two years later, Liu et al., observed a similar effect in fish supplemented with *B. subtilis* E20 isolated from fermented boiled soybeans and then infected with GIV (252). Decreased fish mortality and increased survival rate were observed. In both studies, the administered probiotics also enhanced the innate immune responses (respiratory burst, plasma lysozyme activity, phagocytosis activity and alternative complement activity). Also, dietary supplementation with commercial probiotic named Lactobacil, individually or mixed with Sporolac, in olive flounder naturally infected with lymphocystis disease virus (LCDV) enhanced disease resistance (256). In another study, hulong grouper fed *B. subtilis* 7k were significantly strengthened in innate immune functions when compared with those fed with control diets. Moreover, *B. subtilis* 7k supplementation inhibited infection by Singapore grouper iridovirus (SGIV) (253).

In other cases, probiotic strains have been used as vectors to administer viral antigens to the host. Through this strategy, probiotics would exert all their beneficial effects and be at the same time vaccine vectors. Along this line, a study carried out by Min et al. with rainbow trout orally immunized with *Lactobacillus*-expressing the VP2 and VP3 protein of the infectious pancreatic necrosis virus (IPNV) resulted in reduced viral loads, as analyzed by real-time RT-PCR after IPNV challenge (254). Likewise, oral immunization of rainbow trout with recombinant *L. lactis* NZ3900 expressing the G gene of viral hemorrhagic septicemia virus (VHSV) resulted in a significant reduction of viral loads and decreased fish mortality after viral challenge (255). Increased resistance to IPNV was also detected in rainbow trout orally immunized with recombinant *L. casei* expressing the viral antigens (264). Similar results were observed in common carp orally immunized with recombinant *L. plantarum* expressing the G protein of spring viremia of carp virus (SVCV).

### Probiotics Providing Resistance to Parasites

Although parasitic infections often provoke lower mortalities than viral and bacterial pathogens, they adversely affect animal health, with an enormous impact on aquaculture from an economic point of view. Despite this, not many studies have investigated the capacity of probiotics to confer resistance against different types of parasites (**Table 3**). In one of these studies, Pieters et al. demonstrated that the oral administration of *A. sobria* GC2 and *Brochothrix thermosphacta* BA211 to rainbow trout conferred increased resistance against *Ichthyophthirius multifiliis* (Ich), with the *A. sobria* GC2 strain being more effective in its protecting role (212). In a similar study, dietary supplementation with *B. subtilis* spores expressing *Clonorchis sinensis* paramyosin protected grass carp from cercaria infection (257). In another study, catfish exposed to *L. plantarum* showed a reduced infection with *Saprolegnia parasitica* (259). Finally, in Yanuhar et al. (258), probiotic formulations containing a mixture of *Bacillus* spp., *Lactobacillus* sp. and *Nitrosomonas* sp., were administered at different doses to Koi carp and resulted in increased disease protection against *Myxobolus* sp., in terms of gill tissue damage reduction. Further investigations are needed in fish to explore additional effects of probiotic treatment on the resistance to parasitic infections.

## General Conclusions

The use of probiotics in aquaculture is a promising approach to increase fish health status and reduce the impact of infectious diseases. Reports in the past years, have broadly established that there is a wide range of probiotic microorganisms that can produce beneficial effects to the host, including immunostimulatory effects, increased resistance to pathogens, stimulation of growth, increased digestion or even improved water quality. Despite all these studies, the use of probiotics in aquaculture is not as extended as would be expected taking into account the effort that has been devoted to this research field in the past years. In this sense, we thought of importance to gather in a specific review all direct evidence of increased protection to pathogens conferred by probiotic administration. As visualized in this review, most of the efforts have been directed to establish how probiotics can protect against bacterial infections, but much less is known regarding their antiviral or antiparasitic effects. Nevertheless, probiotic application is a dynamic research field in the sense that there is a continuous search for new probiotic candidates that have even more beneficial effects. Despite the numerous reports on fish probiotics, the mechanisms through which these probiotics exert their effects has not yet been clarified in many cases. Other practical and safety issues also need to be addressed to convince farmers that probiotics are safe and eco-friendly alternatives to chemotherapy. Thus, for each probiotic candidate, the best administration regime should be established, determining for how long these probiotics should be administered to colonize the mucosal surfaces and obtain optimal results. Finally, the safety of each candidate has to be established beyond doubt, including a determination of the antibiotic resistance and a confirmation that any resistance will not be transferred to surrounding microorganisms. Hopefully, all these studies focused on providing insights on the mechanisms of action of probiotics, practical administration issues and safety will contribute to stimulate the regular use of probiotics in aquaculture.

## Author Contributions

RS, CT, and PD-R collected information and wrote the manuscript with help and contributions from FD and NN-O. All authors contributed to the article and approved the submitted version.

## Funding

This work was supported by the European Research Council (ERC Consolidator Grant 2016 725061 TEMUBLYM), by the Spanish Ministry of Science, Innovation and Universities (project AGL2017-85494-C2-1-R) and by the *Comunidad de Madrid* (grant 2016-T1/BIO-1672).

## Conflict of Interest

The authors declare that the research was conducted in the absence of any commercial or financial relationships that could be construed as a potential conflict of interest.
